# Evolving Global Migration Trends: Post-Migration Experiences of Iranian Dentists Attempting to Obtain Credential Recognition in Canada

**DOI:** 10.3390/ijerph22050725

**Published:** 2025-05-02

**Authors:** Sara Hajian, Glen E. Randall

**Affiliations:** 1 Global Health Program, Faculty of Health Sciences, McMaster University, 1280 Main St. West, Hamilton, ON L8S 4M4, Canada; hajiansara@yahoo.com; 2 Health Policy and Management Area, DeGroote School of Business, McMaster University, 1280 Main St. West, Hamilton, ON L8S 4M4, Canada

**Keywords:** global migration, immigration, dentists, integration, mental health

## Abstract

As global migration continues to expand, the diversity of migrant populations increases. This includes a growing number of highly educated individuals from lower-income countries who face significant economic and mental health challenges in attempting to integrate into new communities. Despite extensive education and experience, their expertise is often unrecognized, with many resorting to unskilled labor alternatives. While substantial research exists on the immigration experiences of physicians and nurses, little is known about other professionals, such as dentists. This case study seeks to gain an in-depth understanding of the post-migration experiences of Iranian-trained dentists in Canada, identifying barriers and facilitators to their successful integration. Using a qualitative approach, this study is based on eleven interviews with dentists trained in Iran who recently immigrated to Canada. Semi-structured interviews were conducted via Zoom in English. A thematic analysis was performed using the 2021 Dedoose software program. Barriers to successful integration were categorized into two main themes: “socio-cultural” and “institutional” impediments. The findings show that participants faced significant challenges integrating into Canadian society. Beyond the many socio-cultural obstacles, the negative economic and mental health impacts of attempting to navigate the credential recognition system were substantial, largely due to what appears to be a systematic and institutionalized bias against foreign-trained individuals built into the credentialing system. As a result, their skills often remain underutilized, benefiting neither themselves nor their new country. Findings will inform policy and practice and propose practical recommendations that include reducing institutional barriers for credential assessment, providing mental health support, and offering financial support during assessment of international education.

## 1. Introduction

In recent decades, global immigration has surged, with 281 million people migrating from their home countries in 2020 alone—a stark increase of 60 million compared to 2010 [1]. As global migration continues to expand, the diversity of migrant populations increases [2]. This migration wave includes a substantial number of highly skilled professionals, particularly from Low-and Middle-Income Countries (LMICs) to High-Income Countries (HICs) seeking enhanced career and educational opportunities and an improved quality of life for them and their families [3]. Paradoxically, this trend exacerbates the global healthcare worker shortage, projected to reach 18 million by 2030, due to the emigration of these professionals [4]. Consequently, this migration pattern threatens to accentuate disparities in healthcare resources and access [5].

Notably, nations like the United States, United Kingdom, Canada, and Australia rely heavily on International Medical Graduates (IMGs), who constitute approximately 25% of their physician workforce, mostly originating from LMICs [6]. Canada, renowned for attracting highly skilled workers, saw over 50% of its new immigrants in 2019 holding post-secondary graduate degrees [7]. However, the effectiveness of this talent influx hinges on the successful integration of foreign-trained healthcare workers into their destination countries’ healthcare systems [8]. Despite extensive education and experience, a growing number of highly educated individuals from lower-income countries face significant economic and mental health challenges in attempting to integrate into new communities, and their expertise is often unrecognized, with many resorting to non-clinical or unskilled labor alternatives [9].

Despite its progressive immigration policies, Canada faces integration challenges, as do other countries [10]. Meanwhile, LMICs, like Iran, grapple with the significant loss of educated professionals, including medical graduates [11]. Iran has historically ranked high among LMICs in terms of skilled labor migration, including healthcare workers [12]. Moreover, Iran features among the top ten source countries for permanent residents in Canada [7], particularly concentrated in the Greater Toronto Area [13].

While substantial research exists on the immigration experiences of physicians and nurses, little is known about other professionals, such as dentists. This case study seeks to gain an in-depth understanding of the post-migration experiences of Iranian-trained dentists in Canada, identifying barriers and facilitators to their successful integration. An examination of their migration experiences can unearth gaps and potential improvements in their integration, which is intertwined with the quality of dental care they deliver in their host countries. The findings from this study could not only assist international dental graduates from LMICs in navigating the Canadian migration process but also contribute to the decision-making processes of dental professionals in LMICs considering migration for their future careers. Furthermore, this research can enlighten policymakers in destination countries about the challenges faced by international dentists and provide insights into how their integration can be enhanced. In essence, this study seeks to enrich the field of migration studies by focusing on the specific experiences of Iranian oral health professionals migrating to Canada.

## 2. Materials and Methods

A qualitative exploratory case study was conducted with a holistic approach to gather diverse perspectives and an in-depth understanding of the barriers and facilitators associated with successful integration of Iranian-trained dentists. This methodology is well established and was specifically selected to acknowledge the subjective nature and potentially diverse views of the participants. Virtual face-to-face semi-structured interviews were conducted with eleven Iranian dentists who had migrated to Ontario, Canada. The interview questions, adapted from a previous Australian study [14], covered topics such as the immigration process, social and cultural experiences, integration, study/work life, equivalency credential exams, and licensing processes.

Interviews were conducted via Zoom, adhering to COVID-19 precautions, with a process in place to maintain participants’ privacy and confidentiality. The study population consisted of Iranian IDGs (international dental graduates) residing or working in Ontario, Canada, who met specific study criteria. These criteria included the following: migration more than five years ago, graduation from an Iranian dental school, involvement in the Canadian equivalency examination process, and a self-identified ability to communicate in English. Those not meeting these criteria were excluded.

A purposeful sampling strategy was employed, initially identifying participants through professional social networks and ensuring diversity in terms of gender, age, and years of practice. Recruitment involved making social media posts to a specific site that was dedicated to Iranian-trained dentists who were attempting to navigate the Canadian equivalency process, asking for volunteers to participate in the study. The details of the post received institutional ethics approval. Individuals who expressed an interest in participating in the study were contacted for further personal details, to explain the study and obtain informed consent, and scheduling of the interview. Iranian dentists who migrated to Canada more than five years ago and went through the process to determine equivalency of training and obtain a license to practice dentistry were interviewed between June and September 2021.

Ethical approval was obtained from the McMaster University Research Ethics Board, and informed consent was obtained from each participant. Confidentiality and data security were rigorously maintained throughout the study. Participants had the freedom to decline to answer any questions and could withdraw from the study at any time. Audio-recordings and confidential data were stored securely and anonymously.

To build trust and comfort, informal discussions in Persian (participants’ home country language) were held before the formal interviews began. All interviews were conducted by the same female researcher. Regular meetings with the research team ensured project progress and integrity. The hour-long interviews were recorded digitally and transcribed verbatim. A total of eleven interviews were conducted, and the transcripts were analyzed thematically using the Dedoose software program [15], following Braun et al.’s guideline [16]. Potential themes were initially identified based on a literature review. Following a review of the transcripts from the first three interviews, codes were modified, and additional themes were identified, to make sure subtle patterns not originally included were added to the coding script prior to coding subsequent interviews. Once it was determined that a saturation of views had been achieved and no new themes were identified, no new interviews were conducted. Following the final thematic analysis process, the researchers discussed and reconciled any variations in views. Credibility was enhanced through team debriefing during data analysis, and initial themes related to research questions were generated, reviewed, and refined to ensure validity.

The primary strength of this study was the in-depth understanding of barriers and facilitators to community integration following migration through researcher–participant interaction, which in turn contributed to the high-quality outcome and improved the validity of the study. This enabled the interviewer to build a rapport with the participant and probe statements and underlying assumptions in an informal environment to produce valuable and meaningful data and to essentially coproduce the collection of data. Broad open-ended questions allowed participants to raise issues that were important to them. The interviews were conducted in English to eliminate the possibility of translation bias which could emerge during the interpretation of data.

The weaknesses of this study are mostly related to the limitations caused by the nature of qualitative studies, including potential researcher bias, the potential subjectivity of interviews, and the possibility of respondents withholding information about sensitive issues. In addition, Iranian dentists who were not successful in having their credentials recognized through the examination process were more likely to hesitate in participating because of their concerns about revealing their identity and losing their home country prestige. Therefore, the relatively small sample size of the study could be non-representative of our study population. However, it was clear that the themes identified were consistent across both respondents that were successful in the credential recognition process and those that were not. Additional respondents were not sought following this saturation of themes. The themes and results identified in the study were limited to Iranian dentists and only reflected their perspectives about attempts to integrate in Canada.

## 3. Results

Integration challenges faced by Iranian-trained dentists in Canada include issues that are typically common to all immigrants, but they also highlight those specific to professionals seeking to have their foreign training and qualifications recognized.

Participants included seven females and four males. Nine were between 30 and 40 years old, and each of these were married. Six participants were married to other dentists who were also going through the dental education equivalency process and could, therefore, also provide information on their partners’ experience. Participants also represented the different paths that foreign-trained dentists can take to have their credentials, and their qualifications recognized. Seven participants eventually qualified through the successful completion of direct licensing examinations, two participants attended the degree completion program at the University of Toronto (which is essentially a program to prepare individuals for the credentialing examination), and a third person was about to begin the program. Three participants were still preparing for the examinations, while one of the participants had just failed the examination for a third time. All the participants were living in Ontario and two of them, having eventually completed all credentialing requirements, were working in their own dental offices.

After conducting eleven personal interviews through the Zoom platform, the point of saturation was reached where no significant new information or themes emerged. Two main areas including “integration barriers” and “integration facilitators” were analyzed thematically. The facilitators encompassed both “emotional support” and “professional support”, while the two primary themes of “socio-cultural barriers” and “institutional barriers” constituted the barriers. Socio-cultural barriers were identified as “communication difficulties”, “homesickness”, and “racism”. Institutional barriers of integration were categorized into “pre-licensing”, “licensing”, and “post-licensing” stages. Themes, sub-themes, and codes are summarized in Table 1.

### 3.1. Socio-Cultural Barriers

Socio-cultural challenges encompassed language barriers, experiences of racism and discrimination, and homesickness. Participants highlighted the initial difficulty of communicating in English upon arrival, exacerbated by the lack of adequate English education in Iran. As one participant lamented, “As Iranians, we don’t get to speak English in our community or have proper English education in our home country”. Another participant emphasized the importance of language skills for integration, stating, “If you want to be open to learning different cultures or new things in a new society, you need to improve your language skills”.

The COVID-19 pandemic further hindered social interactions, limiting opportunities to build connections. As one participant mentioned, “It was very difficult for me to speak on the phone, to make an appointment or booking request for personal services. But, I was able to join a volunteer research activity, and it was very useful for me until COVID happened”.

Homesickness was a prevalent theme, with participants expressing a strong emotional attachment to their homeland. One participant described the contrast, saying that “In Iran, they talk in your own language, and you’re raised there, and a strong bond with your homeland is always there”. However, some participants noted that different personalities could affect a person’s experience during migration. One participant mentioned that they faced challenges during their early years in Canada but emphasized the importance of patience and flexibility in overcoming difficulties.

Experiences of racism were reported, though some participants noted supportive communities and patients in their work. One participant experienced racism at a Canadian university, stating that “I am bullied for being different and having a difficult name for them to pronounce”. Another participant mentioned instances of discrimination related to the admission process in some Canadian dental schools, particularly concerning individuals wearing hijabs.

### 3.2. Institutional Barriers

Institutional barriers encompassed challenges related to the recognition of foreign educational documents and employment regulations in Canada. Participants acknowledged the necessity of examinations to verify qualifications and ensure patient safety but described them as difficult and stressful. Institutional barriers were categorized into three sub-categories: pre-licensing, licensing, and post-licensing stages.

During the pre-licensing phase, challenges emerged in document submission and the registration process for dental exams. Delays in document submission, often due to issues like unsealed documents from foreign dental schools, frustrated participants. As one participant recounted, “Some of my documents had to be sent directly from my dental school back in Iran. For some reason, the officer decided to open the sealed documents. So, the dental board declined those unsealed documents. The processing was unreasonably long, and I missed a year to resubmit and register for the exam”.

Securing a spot for the clinical equivalency examinations proved challenging, with limited capacity and quick fill-ups. One participant shared their experience of continuously monitoring the National Dental Examining Board of Canada (NDEB) website to secure an exam date: “One of the biggest challenges in the licensing process is simply securing a spot for the exams. The moment the registration site opens, all available seats are gone within seconds. If you don’t get a spot in your city, you’re forced to travel- sometimes across the country- paying for flights, accommodation, and other expenses just to take the exam. It adds unnecessary stress and financial burden to an already difficult process. It becomes even more stressful when you finally take the exam but then face a long waiting period for your results. During that uncertain time, the registration for the next exam opens, and without your results, you miss out on re-registering. You’re then forced to constantly monitor the website, hoping for a cancellation or a withdrawn spot, all while not knowing whether you should continue studying or if it is a wasted effort…”.

Regarding information on the equivalency process, participants found the NDEB website lacking in detailed guidance. As one participant stated, “The NDEB website is just a standard platform with very brief information. You cannot find different institutes for classes or training courses or the complexity of the process”. Consequently, they relied heavily on unofficial information from friends or peers who had followed a similar path.

While the NDEB offered exam preparation resources, participants found them to be extensive and overwhelming. One participant expressed frustration, saying that “You see a bunch of textbooks and papers on the website that they are using for either AFK [assessment of fundamental knowledge exam] or ACJ [assessment of clinical judgment exam]. But, it takes years to finish them all, and then you still don’t know what kind of exam you’re going through”.

Additionally, the financial burden of exam fees, training courses, and living expenses placed significant stress on participants and their families. Funding support was scarce due to low passing rates, especially in clinical exams. As a participant noted, “Many of my friends had problems passing the clinical exams, while it is a very expensive one. Besides, the professional line of credit from the government or other organizations for international dentists is limited”.

Licensing presented challenges in terms of timing, costs, content, and formats during the equivalency evaluation process. Some participants voiced concerns about the subjectivity and low passing rates in the Assessment of Clinical Judgment (ACJ). One participant highlighted the inconsistency, stating, “It is very subjective, and much depends on the examiner’s opinion. Different dentists may have different opinions on the same case. There is no standardization. Even Canadian dental graduates could face serious trouble in passing it”.

Participants also found the exam content culturally insensitive, as they struggled to understand the Canadian dental system. “In Iran, I hardly worked with CAD [computer aided design to digitally design dental restorations like crowns] or CAM [computer-aided manufacturing to fabricate various restoration items] systems or digital scanners, or some of the advanced implant techniques, that I’m expected to understand and apply these concepts in the exam without any prior hands-on experience”. Or, “Even in pharmacology, the medications and managements are different and complicated. The drug names don’t always match what we learned back home”. Increasing the time allocated for the clinical examination was suggested to improve work quality: “You don’t watch your work just because you don’t have time. If you have to keep the patient longer, you would do that to give the best quality to your work. When it comes to the exam, that’s not given to you, which I think it’s not fair”.

Some participants believed that the exceptionally low passing rates for the equivalency exams were driven by profit motives, viewing the NDEB as a business rather than a regulatory body. One respondent noted, “They intentionally maintain the passing rate very low, while the cost of exams, especially the clinical ones, is high. So, consider how much money is spent by each participant to register and pass these exams. I believe they have benefits in running these exams”. For the clinical exam, candidates must practice on specific artificial teeth that match those used by the NDEB. However, these teeth are difficult to find on the market and come at a high cost, adding another layer of financial and mental strain.

Post-licensing referred to international dental graduates who successfully completed the equivalency process and began practicing as dentists in Canada. Challenges included office availability, costs, a lack of ready-to-practice programs, and an unfamiliarity with the Canadian dental system.

Participants emphasized the importance of workplace skills, particularly “patient management”, as a key difference between Canada and Iran. They noted the need for improved communication with patients and adapting to a more patient-centered approach in Canada. Participants felt that the NDEB process inadequately prepared them for practicing dentistry in Canada, with gaps in education on patient ethics and communication skills. Information on the Canadian dental system was limited on the NDEB website, necessitating reliance on alternative sources.

Navigating the NDEB website posed challenges, with participants sometimes unaware of the need for provincial licensing. Additionally, understanding insurance and other practical aspects of Canadian dentistry took time to grasp.

### 3.3. Integration Facilitators

While much of what the participants described would be classified as barriers to integration, there were a few examples provided of emotional and professional support systems that facilitated their integration.

Emotional support from family, friends, and the Iranian community in Canada played a crucial role. One participant emphasized the value of family support, saying that “When I moved to Canada, my fiancé was already in Canada. I had a very strong emotional support and a solid source of information”. Another participant appreciated the support from the Iranian community, stating that “They (Iranian community) gave me some useful information about where to live, where to go, where to get my groceries, how to get, for example, my health card, or opening a bank account”.

Professional support included attending training courses, especially those run by Iranian dentists, to gain insight into exam formats and content. One participant highlighted the importance of these courses, saying that “When I went to preparation courses, at least they told me how the exams look like, or what kind of questions will be asked in the exams”. Participants also stressed the need for opportunities to shadow local dentists and acquire hands-on experience in the Canadian dental system.

Attending social events, taking English courses, and building peer networks were additional strategies to enhance integration. Participants believed that being open-minded and welcoming to different cultures were essential for successful integration. Peer networks and contacting Iranian IDGs who had gone through a similar path helped most participants collect useful information about navigating the process and exchanging information about the content of the exams. One participant emphasized the value of attending preparation courses, especially those run by Iranian dentists, stating that “You can find many Iranian internationally trained dentists taking the course. If you go there, you can find a lot of friends, and they can help you get through the process”.

## 4. Discussion

This qualitative study investigated the post-migration experiences of Iranian dental graduates in Canada. This research revealed that some Iranian dental graduates had experienced significant challenges with communication, practicing within the job setting in the Canadian context, and the process for evaluating equivalency of qualifications. Participants described mostly relying on their ethnic and peer networks to navigate the Canadian system while experiencing emotional and financial stress, mainly at the time of arriving in Canada and during the preparation for the NDEB examinations. Family and peer support helped them cope with the various challenges they faced. These findings are consistent with previous studies that underscore the importance of social networks and familial support in easing the adaptation process for immigrants in the healthcare professions [17].

The generally multicultural, migrant-friendly nature of Canada and the existence of a large Iranian community improved integration experiences for Iranian dentists as they reported low or no cultural shock at the time of arriving in Canada. Yet, a few of them pointed out what they perceived as undercurrents of racism, mainly with regard to their difference in appearance. While one respondent commented on reactions to wearing a hijab, there was a surprising lack of overt comments regarding religion or feeling persecuted because of religious beliefs. However, similarly to another study’s findings, participants had expected to experience some level of discrimination, and they accepted or ignored it in order to focus on their career path [18]. Such resilience against discrimination aligns with other studies suggesting that immigrants often display psychological flexibility in adjusting to cultural differences, which allows them to prioritize professional aspirations over societal biases [19]. Iranian dentists, while acknowledging discrimination, also viewed it as something they could overcome as they pursued their professional goals [20].

The main social barrier to integration mentioned by all participants was the language barrier. Consistently with other studies, difficulties in understanding other nationalities’ accents, routine slang, and culture could directly affect social communication and adaptation for participants, even in their dental practice [14,21]. Furthermore, previous research has emphasized the complex role of language proficiency in professional integration, where a lack of fluency can contribute to feelings of exclusion, even among highly qualified professionals [22]. These findings underscore the critical need for targeted language support programs for immigrant healthcare professionals in Canada.

Institutional barriers during different stages of dental licensing were the most crucial challenges faced by Iranian-trained dentists in Canada. The NDEB equivalency examinations were perceived to be tough and stressful, but more importantly, unfair, biased, not culturally sensitive, and unnecessarily lengthy. The low success rate on first attempts made some participants wonder whether there were any underlying financial motives at play. Similarly to the New Zealand Dental Registration Examination and Australian Dental Council Examination, several challenges were reported about the lack of proper information on the exams’ contents and preparation courses, and the need for support systems [22]. This resonates with other studies that highlight the opaque nature of licensing exams in various countries, which can disproportionately affect internationally trained professionals [23]. Furthermore, respondents’ perceptions of a lack of transparency in licensing processes, has been reported to increase stress and reduce the sense of fairness, which can negatively impact the overall integration process for immigrant professionals [24].

Moreover, the lack of a resident Canadian embassy in Iran due to political conflicts and poor communication between dental schools in the two countries made the registration process even more challenging for Iranian dentists. The NDEB and Canadian dental schools could facilitate this process by introducing alternate ways for Iranian dentists to submit their educational documents. Studies have emphasized the critical role of administrative support in facilitating the professional integration of internationally trained healthcare workers [10].

Meanwhile, the new strategic plan presented by the NDEB in October 2021 presents some structural changes in the format of exams assessing the competency of dental graduates of non-accredited dental schools. The ACS [assessment of clinical skills exam] was proposed to be replaced by a new exam called the National Dental Examination of Clinical Competence (NDECC) in June 2022 [25]. The plan was to offer it multiple times a week with an unlimited number of times to attend within a five-year period instead of a maximum of three attempts at the ACS [25]. This test consists of “clinical skills” and a new component of “situational judgment” and will only be conducted in Ottawa, in which all required materials and instruments are provided, and permanent residents or citizens are given priority to take the exam [25]. These various changes announced by the NDEB appear to be an acknowledgement of some of the negative perceptions about the exam and its processes as identified in this study. The changes seem to represent a move toward a more flexible and responsive assessment process [25]. Future research needs to evaluate and compare IDGs’ experiences regarding these recent changes.

Overall, the obstacles Iranian-trained dentists face in integrating into Canadian society are perceived by respondents to be mostly due to institutional barriers, including the lengthy process of licensing, non-culturally sensitive questions, limited access to exams, difficulty in submitting required documents, and the costs of the exams. In addition to these findings, difficulties in entering dental university programs and passing the clinical examinations on the first attempt raise a question regarding potential systematic bias against foreign-trained dentists in Canada. While the main role of the regulatory college and the exam board is to fairly assess the qualifications of foreign applicants, respondents uniformly suggested that they tend to keep out foreign-trained dentists, including Iranian-trained dentists, for several intentional or unintentional reasons. Behind the scenes, respondents noted that the process is unreasonably difficult for IDGs, maybe due to saving jobs for domestic dentists, making money, or systematic discriminatory attitudes.

This perception, real or not, could adversely affect Iranian-trained dentists’ mental health and put additional pressure on their families. Regulatory practices and orientation courses on new professional and cultural environments could help them better integrate and communicate. Further, Iranian dentists argue for change in the process of licensing and navigating the process. They suggest the system needs to be more equitable for them, not specifically easier. Although this study underrepresents the number of respondents who were unsuccessful in completing the equivalency process, The results are not likely to change as there were not notable variations in views between those were successful and those that were unsuccessful in the equivalency process. In the absence of changes and a comprehensive approach in the Canadian system, IDGs may continue to feel challenged and stressed as they attempt to navigate the Canadian system.

This article focuses primarily on the direct licensing pathway through NDEB, as opposed to the university route, which has evolved significantly. Canadian universities now require a different exam called the Advanced Dental Admission Test (ADAT), and each institution has its own requirements, with limited positions available for accepting international dentists into their programs. This adds another layer of complexity for those seeking to practice in Canada, as competition for spots is fierce and the process can be lengthy and costly, making the direct licensing process through NDEB the more straightforward option for many internationally trained dentists.

Beyond the specific challenges faced by these respondents, findings raise broader questions about systemic barriers that may be unintentionally raised through immigration policies, professional credentialling, and in general, inadequate workforce planning. In particular, these barriers may have a negative impact on efforts to promote equity and sustainability. Recent studies underscore the significance of migration policies that tackle systemic credentialing issues, pointing out the unintended negative impacts of strict accreditation systems on sustainable workforce planning. For instance, Moyo et al. note that licensing restrictions can cause the deskilling of immigrant professionals, leading to economic inefficiencies and the loss of valuable human capital [26].

Our results reinforce concerns that various barriers can delay the integration of skilled labor from other countries, or in some instances, block the full utilization of these professionals all together. The result is the loss of opportunities for immigrants and the inefficient use of human capital. Likewise, Aniche et al. stress that the relationship between migration and sustainable development is closely linked and they therefore highlight the need for effective management of migration to fully realize its potential benefits [27]. These insights highlight the necessity of reforming credentialing processes to ensure skilled professionals can seamlessly integrate into host labor markets.

Although it is beyond the scope of this current paper, future work in this area may benefit from a more theoretically driven approach to demonstrating the connections between specific types of migration, political economy, and sustainable development [28].

## 5. Conclusions

Following migration, Iranian dental graduates face several challenges, mainly in terms of institutional barriers in order to become licensed to practice dentistry in Canada. The main arguments emerging are about the lack of support, both financially and mentally, as well as the knowledge gap regarding the equivalency process and exams. The findings also indicate deficiencies in the NDEB process of evaluating qualifications for IDGs graduated from non-accredited dental schools. They have to rely on costly training courses, or they struggle to find a local mentor to acquire the necessary skills and increase their chances of success. It seems female dentists, specialists, and older Iranian IDGs experienced more challenges with the examinations and practice of dentistry in Canada, which indicates the need for intersectionality-based analysis and research in the future.

In summary, immigrant dental graduates from Iran are being needlessly stressed and there is a waste of talent for those who are not deemed to have equivalent professional qualifications. It is difficult to be sure about the true motives behind the process of qualification due to the current systematic bias which disadvantages IDGs in different ways. At a minimum, it should be further investigated to identify exam board and regulatory college motivations behind the equivalency evaluation process. There is still a long way to go in order to see true equity between domestic dentists and international dentists in terms of qualifying for license to practice. It would be helpful to lower institutional barriers and provide equitable access for IDGs, including Iranian dentists, in Canada. Canadian authorities should proactively collaborate with the NDEB authorities to help them minimize the adverse effects of “Brain Waste”. In fact, migration is a common emerging phenomenon, and international collaboration is needed to control its consequences, both in source and destination countries.

## Figures and Tables

**Table 1:** Barriers and facilitators to integration.

Domains	Themes	Sub-Themes	Codes
Integration Barriers	Socio-Cultural Barriers	Communication difficulties	Language barrier
			Lock-down policies
		Homesickness	Having friends or family in Canada
			Personality type, personal attitudes
		Racism	University instructors
			University admission criteria
			Applying for a job
	Institutional Barriers	Pre-licensing	Submitting documents
			Registration Process
			Lack information
			Timing
		Licensing	Content of exams and non-culturally sensitive questions
			Subjective judgment
			Costs of exams and lack of funds
			Timing
			Resources and references
			Low passing rates and limitations on accessing the exams
		Post-licensing	Office availability
			Patient management skill
			Lack of information about provincial licensing
			Costs
			No ready-to-practice program
Integration Facilitators	Emotional Support	Ethnic networks	Navigating the settling down process
			Finding friends and being open-minded
		Family coordination	The role of woman and setting priorities for timing of the exams
			Family ties and financial supports
	Professional Support	Training courses	Private institutions for exam preparation
			Being mentored by local dentists or employed as a dental assistant
			Taking university courses or being involved in research activities
		Peer networks	Navigating the system
			Using the informal knowledge and experience about the equivalency and licensing process

## Data Availability

The data presented in this study are available upon reasonable request from the corresponding author.

## Abbreviations

The following abbreviations are used in this manuscript:

LMIC	Low-and Middle-Income Countries
HIC	High-Income Countries
NDEB	National Dental Examining Board of Canada
IMG	International Medical Graduate
IDG	International Dental Graduate
AFK	Assessment of Fundamental Knowledge exam
ACJ	Assessment of Clinical Judgment exam
ACS	Assessment of Clinical Skills exam
NDECC	National Dental Examination of Clinical Competence
ADAT	Advanced Dental Admission Test

## References

[1] United Nations Department of Economic and Social Affairs International Migration 2020 Highlights.

[2] World Economic Forum Barriers to Migration. Global Risks Report 2022.

[3] Aluttis C., Bishaw T., Frank M.W. (2014). The workforce for health in a globalized context: Global shortages and international migration. Glob. Health Action.

[4] World Health Organization (2019). Addressing the 18 Million Health Worker Shortfall—35 Concrete Actions and 6 Key Messages.

[5] Balasubramanian M., Brennan D.S., Spencer A.J., Short S.D. (2016). The international migration of dentists: Directions for research and policy. Community Dent. Oral Epidemiol..

[6] Zurn P., Dal Poz M.R., Stilwell B., Adams O. (2004). Imbalances in the health workforce: Assessment of the health workforce requirements for universal health coverage and the impact of migration. Hum. Resour. Health.

[7] Immigration, Refugees and Citizenship Canada Express Entry Year-End Report 2019. https://www.canada.ca/content/dam/ircc/migration/ircc/english/pdf/pub/2019-ee-report-pdfversion-eng.pdf.

[8] Lofters A., Slater M., Fumakia N., Thulien N. (2014). “Brain drain” and “brain waste”: Experiences of international medical graduates in Ontario. Risk Manag. Healthc. Policy.

[9] Balasubramanian M., Brennan D.S., Spencer A.J., Short S.D. (2015). The ‘global interconnectedness’ of dentist migration: A qualitative study of the life-stories of international dental graduates in Australia. Health Policy Plan..

[10] Covell C.L., Neiterman E., Bourgeault I.L. (2016). Scoping review about the professional integration of internationally educated health professionals. Hum. Resour. Health.

[11] Asadi H., Ahmadi B., Nejat S., Akbari Sari A., Garavand A., Almasian Kia A., Khajeh M. (2018). Factors influencing the migration of Iranian healthcare professionals: A qualitative study. PLoS ONE.

[12] Chaichian M. (2011). The new phase of globalization and brain drain: Migration of educated and skilled Iranians to the United States. Int. J. Soc. Econ..

[13] Gharakhlou M., Langlois L. (2006). Iranian immigrants in the Greater Toronto Area: Settlement patterns and challenges. Can. Ethn. Stud..

[14] Balasubramanian M., Brennan D.S., Spencer A.J., Short S.D. (2016). ‘Newness-struggle-success’ continuum: A qualitative examination of the cultural adaptation process experienced by overseas-qualified dentists in Australia. Aust. Health Rev..

[15] Dedoose. https://www.dedoose.com.

[16] Braun V., Clarke V. (2006). Using thematic analysis in psychology. Qual. Res. Psychol..

[17] Chou L., Kuo S. (2013). The role of social networks and familial support in immigrant adaptation: Implications for healthcare professionals. J. Immigr. Minor. Health.

[18] Davda L.S., Gallagher J.E., Radford D.R. (2018). Migration motives and integration of international human resources of health in the United Kingdom: Systematic review and meta-synthesis of qualitative studies using framework analysis. Hum. Resour. Health.

[19] McLarnon M.J.W., Rothstein M.G., King G.A., Medianu S., Sutter A., Kisinger K. (2023). The effect of resiliency and self-regulation on immigrants’ job search behavior. J. Employ. Couns..

[20] Hajian S., Jadidfard M.-P., Yazdani S., Randall G.E., Khoshnevisan M.-H. (2023). Understanding why oral health professionals migrate: A qualitative investigation of Iranian dentists who have moved to Canada. Glob. Health Action.

[21] Ayers K.M., McMillan A.S., McGrath C. (2008). Understanding foreign-accented English. J. Am. Dent. Assoc..

[22] Sennett R. (2008). The Culture of the New Capitalism.

[23] Archer J., Lynn N., Coombes L., Roberts M., Gale T., Price T., Regan de Bere S. (2016). The impact of large scale licensing examinations in highly developed countries: A systematic review. BMC Med. Educ..

[24] Al Achkar M., Dahal A., Frogner B.K., Skillman S.M., Patterson D.G. (2023). Integrating Immigrant Health Professionals into the U.S. Healthcare Workforce: Barriers and Solutions. J. Immigr. Minor. Health..

[25] National Dental Examining Board of Canada (NDEB) Introducing the NDECC: The Modified Skills Examination. https://ndeb-bned.ca/2021/10/29/introducing-the-ndecc-the-modified-skills-examination.

[26] Moyo I., Nshimbi C.C., Laine J.P. (2020). Migration Conundrums, Regional Integration and Development: Africa-Europe Relations in a Changing Global Order.

[27] Aniche E.T., Moyo I., Nshimbi C., Laine J. (2020). Migration and Sustainable Development: Challenges and Opportunities. Migration Conundrums, Regional Integration and Development.

[28] Manioudis M., Meramveliotakis G. (2022). Broad strokes towards a grand theory in the analysis of sustainable development: A return to the classical political economy. New Political Econ..

